# The roles of tumor necrosis factor-alpha in colon tight junction protein expression and intestinal mucosa structure in a mouse model of acute liver failure

**DOI:** 10.1186/1471-230X-9-70

**Published:** 2009-09-22

**Authors:** Hong-Li Song, Sa Lv, Pei Liu

**Affiliations:** 1 Department of Infectious Diseases, The First Affiliated Hospital, China Medical University, Shenyang 110001, Liaoning Province, PR China; 2 Current address: Department of Organ Transplantation, Tianjin First Central Hospital, Tianjin, 300192, PR China; 3 Current address: The 5th Department of Infectious Disease, 302 Military Hospital of China, Beijing 100039, PR China

## Abstract

**Background:**

Spontaneous bacterial peritonitis (SBP) is a common clinical disease and one of the most severe complications of acute liver failure (ALF). Although the mechanism responsible for SBP is unclear, cytokines play an important role. The aim of this study was to investigate the effects of tumor necrosis factor-alpha (TNF-α) on the structure of the intestinal mucosa and the expression of tight junction (Zona Occludens 1; ZO-1) protein in a mouse model of ALF.

**Methods:**

We induced ALF using D-galactosamine/lipopolysaccharide (GalN/LPS) or GalN/TNF-α and assessed the results using transmission electron microscopy, immunohistochemistry, Western blotting, ELISA and real-time quantitative PCR. The effects of administration of anti-TNF-α IgG antibody or anti-TNF-α R1 antibody before administration of GalN/LPS or GalN/TNF-α, respectively, on TNF-α were also assessed.

**Results:**

Morphological abnormalities in the intestinal mucosa of ALF mice were positively correlated with serum TNF-α level. Electron microscopic analysis revealed tight junction (TJ) disruptions, epithelial cell swelling, and atrophy of intestinal villi. Gut bacteria invaded the body at sites where TJ disruptions occurred. Expression of ZO-1 mRNA was significantly decreased in both ALF models, as was the level of ZO-1 protein. Prophylactic treatment with either anti-TNF-α IgG antibody or anti-tumor necrosis factor-a receptor1 (anti-TNF-α R1) antibody prevented changes in intestinal tissue ultrastructure and ZO-1 expression.

**Conclusion:**

TNF-α affects the structure of the intestinal mucosa, decreases expression of ZO-1, and affects the morphology of the colon in a mouse model of ALF. It also may participate in the pathophysiological mechanism of SBP complicated to ALF.

## Background

Acute liver failure (ALF) is a devastating disease associated with high mortality. Spontaneous bacterial peritonitis (SBP), a common clinical disease, is one of the most severe complications of ALF and a major cause of death [1-4]. However, the mechanism responsible for SBP is unclear. Previous studies reported that the serum level of tumor necrosis factor-α (TNF-α) is elevated in patients with severe liver injury and is positively associated with serum lipopolysaccharide (LPS) level [5,6]. TNF-α is a cytokine with broad-spectrum physio-and patho-responsiveness and is primarily secreted by monocaryons and macrophages. In addition to participating in humoral and cellular immune responses, TNF-α also plays an important role in diseases such as severe hepatitis, septic shock, and inflammatory bowel disease [7-10]. However, it is not known whether TNF-α affects the barrier function of the intestinal mucosa.

The intestinal mucosa is a physical and metabolic barrier against toxins and pathogens in the lumen of the gut. Tight junctions (TJs) are the main structures responsible for restricting paracellular movement of compounds across the intestinal mucosa. Structurally, TJs are composed of cytoplasmic proteins, including the zona occludens proteins, ZO-1, ZO-2, and ZO-3 [11,12] and two distinct transmembrane proteins, occludin and claudin [13,14], which are linked to an actin-based cytoskeleton [15]. TJs function as occluding barriers by maintaining cellular polarity and homeostasis and by regulating the permeability of paracellular spaces in the epithelium [16]. ZO-1, a member of the MAGUK family of proteins, acts as a scaffold for organizing transmembrane TJ proteins and recruits various signaling molecules and the actin cytoskeleton to the TJs [17]. Although previous studies have afforded an insight into the molecular structure of TJs, much less is known about TJ functionality under physiological or pathophysiological conditions. Few studies have described intestinal mucosa ultrastructure or changes in TJs during liver failure. In this study, we used ALF animal models to investigate the effect of TNF-α on the ultrastructure of the intestinal mucosa with emphasis on the role of TJs.

## Methods

### Animals and treatment

Male, six-to eight-week-old BALB/c mice (China Medical University) were obtained from the China Medical University (Shenyang, China). They were housed and cared for in rooms maintained at a constant temperature and humidity. Food and water were allowed ad libitum. Food was withdrawn the evening before the experiment. All animal experimental procedures were approved by the Ethics Committee of China Medical University before the commencement of the study.

All mice were randomly divided into eight groups (n = 8 per group). One group of mice was given intraperitoneal injections of D-galactosamine (GalN; 800 mg/kg body weight; Sigma, Saint Louis, USA) and LPS (10 μg/kg body weight; Sigma) to induce ALF. A second ALF-induction group was also given intraperitoneal injections of GalN (800 mg/kg body weight) and TNF-α (10 μg/kg body weight; Sigma). Two groups were given antibody treatments prior to ALF induction: one was given anti-TNF-α IgG (100 μg per mouse; US Biological, USA) and the other was given anti-TNF-α R1 antibody (100 μg per mouse; R&D Systems, USA). The anti-TNF-α IgG and anti-TNF-α R1 antibodies were injected via the vena caudalis 30 minutes and 15 minutes before GalN/LPS administration. There were four control groups, which were injected intraperitoneally with GalN, LPS, TNF-α, or NS.

In summary, the eight groups were: 1) GalN/LPS; 2) GalN/TNF-α; 3) GalN control; 4) LPS control; 5) TNF-α control; 6) NS control; 7) anti-TNF-α IgG and GalN/LPS; and 8) anti-TNF-α R1 antibody and GalN/LPS. Mice in the first six aforementioned groups were euthanized 2, 6, 9, 12 and 24 h after treatment. Mice in the last two of the aforementioned groups were euthanized 9 h after administration of GalN/LPS. The study was approved by the Ethics Committee of China Medical University.

### Serum TNF-a assay

Serum levels of alanine transaminase (ALT) were determined using an automatic analyzer (Hitachi, Japan). Serum levels of TNF-α were determined using an ELISA kit (R&D Systems) according to the manufacturer's protocol.

### Detection and observation of intestinal mucosal ultrastructure

Ultrathin (70 nm) intestinal sections were examined using a transmission electron microscope (Hitachi H-600, Japan).

### Immunohistochemical detection of ZO-1 in frozen tissue sections

Frozen intestinal tissue sections (5 μm thick) were fixed on glass slides by incubating them in acetone for 10 min at 4°C. The slides were incubated with 3% H_2_O_2 _for 20 minutes at room temperature and indirectly immuno-labeled using an ABC kit (Takara, Japan) according to the manufacturer's instructions. Slides were then blocked in goat serum for 30 min at 37°C and incubated with a rabbit anti-mouse polyclonal ZO-1 antibody (dilution, 1:50; Santa Cruz Biotechnology, USA) at 4°C overnight. For the negative controls, the primary antibody was replaced with PBS. This incubation was followed by incubation with biotinylated goat anti-rabbit IgG (Histostain-Plus kit, ZYMED) diluted 1:300 in PBS for 2 h at room temperature. Sections were rinsed in PBS and then in distilled water. The slides were stained with 3, 3'-diaminobenzidine and counterstained with hematoxylin.

### Western blot analysis of tissue ZO-1 content

Intestinal tissue samples were homogenized in lysis buffer (20 mM Tris-HCl [pH 7.5], 1% Triton X 100, 0.2 M NaCl, 2 mM EDTA, 2 mM EGTA, 1 M DTT and 2 M aprotinin). Proteins (50 μg) were electrophoresed using SDS-PAGE (8%) and transferred to a nitrocellulose membrane. Membranes were blocked with non-fat dried milk in TBS containing 0.05% Tween-20 (TTBS) for 1 h at room temperature and incubated with a rabbit anti-mouse polyclonal ZO-1 antibody (diluted 1:400; Santa Cruz Biotechnology) at 4°C overnight. After three washes in TTBS, the membranes were reacted with a 1:2000 dilution of alkaline phosphatase-labeled goat anti-rabbit IgG (Santa Cruz Biotechnology) for 2 h at room temperature. The immunoreaction was visualized using α-dianisidine and β-naphthyl acid phosphate (Sigma, USA).

### RNA isolation and real-time quantitative PCR

Total RNA was isolated from intestinal tissues using TRIzol Reagent (Invitrogen, USA). RNA was purified using DNase I and depurified using PI-PCI-EHCO. SYBR-green-based real-time PCR (TaKaRa SYBR RT-PCR kit, Japan) was used to measure relative gene expression in each sample. First, we prepared an RNA standard (forward 5'-TTCCGGGTCGTGGATACTT-3', reverse 5'-GTTCCCAGCTTATGAAAGGGTT-3', amplicon size 327 bp) and determined standard curves for the ZO-1 gene and a house-keeping gene (GAPDH RNA standard forward 5'-CAGCCGCATCTTCTTGTG-3', reverse 5'-AGGAGCGAGACCCCACTAA-3', amplicon size 335 bp). PCR was performed using Taq DNA polymerase (Qiagen, Valencia, USA) and oligonucleotide primers for mouse ZO-1 (forward 5'-CGAGGCATCATCCCAAATAAGAAC-3', reverse 5'-TCCAGAAGTCTGCCCGATCAC-3', amplicon size 97 bp) and glyceraldehyde 3-phosphate dehydrogenase (GAPDH; forward 5'-AAATGGTGAAGGTCGGTGTG-3', reverse 5'-TGAAGGGGTCGTTGATGG-3', amplicon size 108 bp). PCR conditions were as follows: one cycle at 95°C for 30 minutes followed by 45 cycles of PCR amplification, each consisting of 95°C for 5 s and 60°C for 20 s. The concentration of mRNA was calculated according to the standard curve and then normalized to that of GAPDH.

### Statistical analysis

SPSS version 10.0 Software was used to perform the statistical analyses. All data were analyzed using analysis of variance (ANOVA) followed by a least-squares difference test. *P *values < 0.05 were considered significant. All data are presented as the mean ± SE.

## Results

### The effect of TNF-α in mice with GalN/LPS-induced ALF

**Figure 1:**
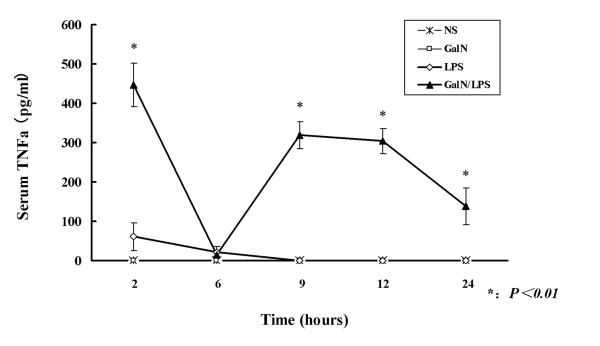
**Serum TNF-α levels**. Serum TNF-α levels increased significantly in GalN/LPS-treated mice. Serum TNF-α levels were determined by ELISA and the data are expressed as the group mean ± SE (eight mice per group).

Most ALF mice (66.7%, 90/120) died between 6 h and 12 h after the GalN/LPS injection. Serum ALT levels increased significantly 6 h after the GalN/LPS injection compared with the control groups (*P *< 0.01) (Table 1). Serum TNF-α levels reached a maximum value (446.18 ± 55.49 pg/ml) 2 h after the GalN/LPS injection and then decreased to 14.82 ± 9.02 pg/ml at 6 h after the GalN/LPS injection (Figure 1). The second highest TNF-α level was observed 9 h after the GalN/LPS injection (319.43 ± 33.72 pg/ml). Liver histopathology showed massive or submassive necrosis at 9 h after the GalN/LPS injection. We successfully used TNF-α in place of LPS to induce ALF in conjunction with GalN. The ALT levels and histopathological characteristics of the GalN/TNF-α group were similar to those of the GalN/LPS group. As the ALF mice that died 9 h (60%, 54/90) after GalN/LPS administration displayed levels of serum biochemical markers and liver morphology consistent with liver failure, we assessed the protective effects of anti-TNF-α IgG and anti-TNF-α R1 antibodies on liver failure 9 h after induction of ALF. The mortality of mice treated with either of the two antibodies was 0% (0/8). ALT serum levels were only slightly elevated and decreased rapidly compared with GalN/LPS-treated mice (*P *< 0.01). Histopathological examination showed only spot or focal hepatonecrosis.

**Table 1:** Serum ALT levels in ALF mice

		2 h	6 h	9 h	12 h	24 h
	
Groups	Number of mice	ALT	ALT	ALT	ALT	ALT
		(U/L)	(U/L)	(U/L)	(U/L)	(U/L)
NS	8	30.0 ± 0.3	29.3 ± 1.0	36.8 ± 2.3	32.5 ± 0.7	30.7 ± 0.4
LPS	8	29.4 ± 0.8	27.1 ± 2.0	40.7 ± 7.0	34.5 ± 3.4	29.1 ± 1.7
GalN	8	44.2 ± 0.4	63.4 ± 1.0	110.1 ± 2.5	97.4 ± 1.2	164.1 ± 10.6
GalN/LPS	8	22.2 ± 2.8	2513.2 ± 874.2^a^	6235.5 ± 912.4^a^	10215.8 ± 967.7^a^	10250.6 ± 1045.8^a^
TNF-alpha	8	34.3 ± 3.4	32.7 ± 4.6	33.4 ± 2.1	34.6 ± 3.9	30.9 ± 8.8
GalN/TNFalpha	8	33.7 ± 2.9	204.1 ± 82.1^a^	4774.8 ± 1118.0^a^	6177.8 ± 1280.9^a^	4204.6 ± 1118.6^a^
anti-TNF-alpha-IgG+GalN/LPS	8	--	--	257.1 ± 83.2^b^	--	--
anti-TNF-alpha R-IgG+GalN/LPS	8	--	--	907.3 ± 551.6^b^	--	--

All values are expressed as mean ± SE. a: *P *< 0.01 vs Saline group, b: *P *< 0.01 vs GalN/LPS co-injection group

### Ultrastructural characteristics of the intestinal mucosa

**Figure 2:**
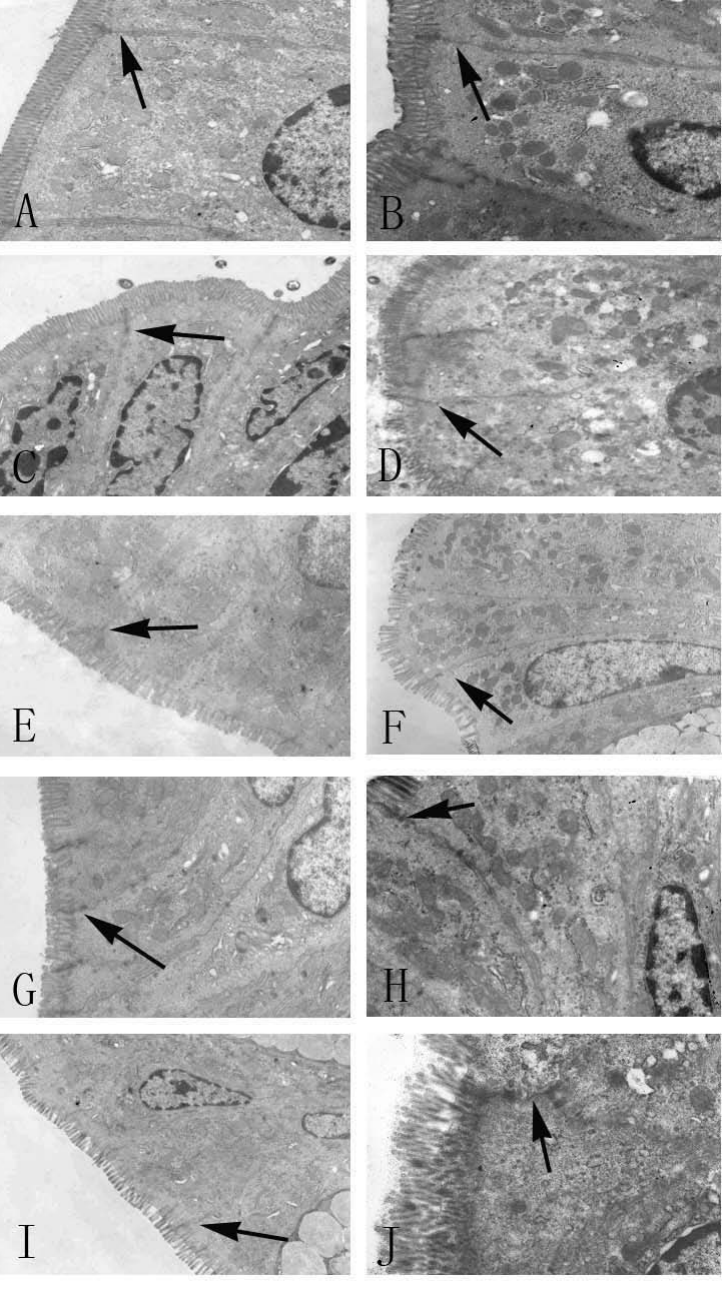
**Transmission electron microscopy of mouse intestine**. Transmission electron microscopy of mice intestine from the control groups (A, E, and F), the GalN/LPS group (B, C, and D), and groups that received antibodies prior to ALF induction (G and H). A) Saline control group (×30,000). Epithelial cells and TJ (→) were intact. B) At 2 h after injection (×120,000). Epithelial cells were swollen and shrunken. Microvilli and organelles were normal. TJs (→) were intact. C) At 6 h after injection (×100,000). Microvilli were almost normal TJs (→) visible in this section were not disrupted. D) At 9 h after injection (×60,000). The mitochondria of the endothelial cells were loose. TJs (→) were disrupted. Organelles were swollen and had reduced electron density. At 9 h after injection (×10,000), some microvilli were loose. A TJ (→) was disrupted. E) LPS control group (×120,000). F) GalN control group (×20,000). Epithelial cells were slightly shrunken and TJs (→) were intact. G) Anti-TNF-α IgG group (×80,000). Epithelial cells were slightly shrunken and TJs (→) between endothelial cells were intact. H) Anti-TNF-α R1 antibody group (×80,000). The TJs (→) between the endothelial cells were intact. I) TNF-α-treated group (×50,000). TJ (→) were intact. J) At 9 h after GalN/TNF-α administration (×100,000). TJs (→) were disrupted.

We observed obvious ultramicrostructural changes in the intestinal mucosa after GalN/LPS administration. Some epithelial cell microvilli were disarranged and distorted, and they were sparsely distributed. The epithelial cells were swollen or shrunken. The mitochondrial matrices were swollen, cristae were breaked and the TJs were disrupted. The changes in the intestinal mucosa of mice treated with GalN/TNF-α were similar to those of mice treated with GalN/LPS. Some TJs were disrupted in the treatment groups, but there was no disruption of TJs in the control groups, only swelling of epithelial cells. Pathological changes in groups that were treated with antibodies were less severe than those in the ALF groups (Figure 2).

### Bacterial invasion of the intestinal mucosa

**Figure 3:**
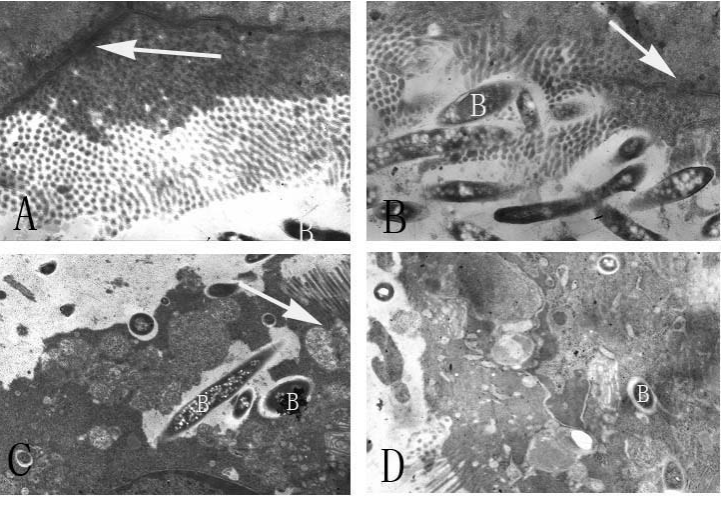
**Morphology and bacterial invasion of colon samples in the GalN/LPS-treated group**. TJs through which bacterial invasion occurred (white color, B) were observed A: 2 h (×100,000), B: 6 h (×120,000), C: 9 h (×60,000), and D: 12 h (×80,000) after GalN/LPS administration respectively. The microvilli (M) were disrupted and the TJs through which bacterial invasion occurred were disrupted at 6 h(B), 9 h(C), and 12 h (D) after GalN/LPS administration. The TJs were disrupted or absent at 9 h(C) and 12 h (D) after GalN/LPS administration.

Under TEM showed that bacterial invasion of the intestinal mucosa began between 6 h and 9 h after GalN/LPS administration. No bacterial invasion was observed in the control groups. The bacteria invaded at sites at which TJs were disrupted. It was evident that bacterial invasion of the intestinal mucosa of the GalN/LPS group occurred via pinocytosis, but this phenomenon was not observed in the other groups (Figure 3).

### Expression of ZO-1 protein

**Figure 4:**
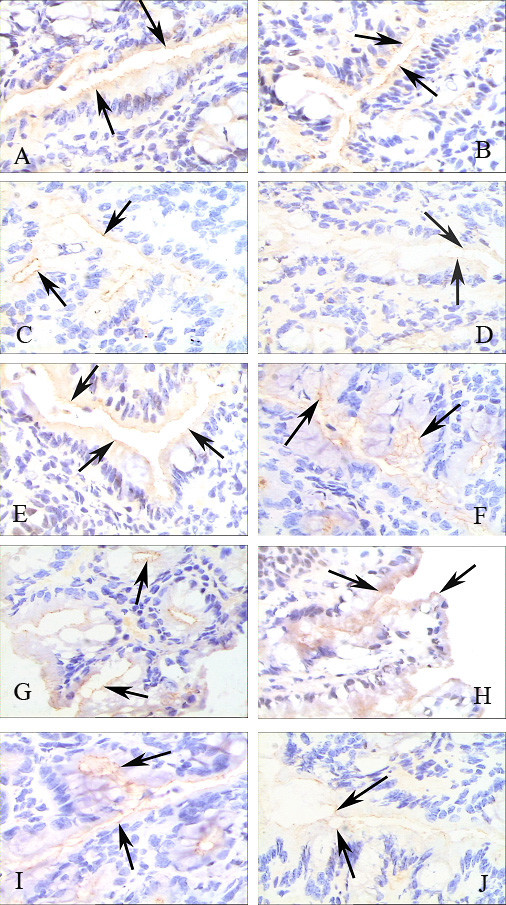
**TJ protein expression in epithelial cells during ALF (original magnification, ×400)**. A: NS-treated group, B: 2 h after GalN/LPS administration, C: 6 h after GalN/LPS administration, D: 9 h after GalN/LPS administration, E: LPS-treated group, F: GalN-treated group, G: anti-TNF-α-treated group, H: anti-TNF-α R1-treated group, I: TNF-α-treated group, J: 9 h after GalN/TNF-α administration. The mucosal tissue sections were double labeled for ZO-1 (brown color). Labeled sections were analyzed immunohistochemically. Decreased ZO-1 staining in the epithelial cells was observed at 9 h (D) after GalN/LPS and GalN/TNF-α (J) administration. In contrast to ZO-1 expression in colon tissue 2 h (B) and 6 h (C) after GalN/LPS administration, ZO-1 expression in the NS group (A), LPS group (E), D-GalN group (F), anti-TNF-α IgG group (G), anti-TNF-α receptor1 group (H), and TNF-α group (I) were not down-regulated (arrows indicate ZO-1).

**Figure 5:**
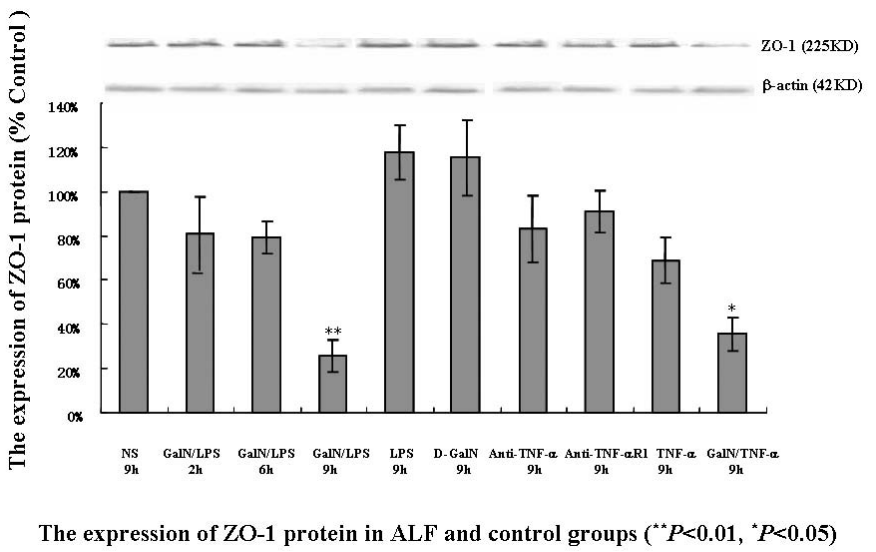
**Mucosal colonic tissues from control and ALF mice were analyzed for ZO-1 expression**. Western blot analyses indicated that ZO-1 expression was altered in mice with ALF. ZO-1 expression was significantly decreased at 9 h in GalN/LPS-treated mice (20.11 ± 7.37%) and GalN/TNF-α-treated mice (35.47 ± 7.34%) compared with the NS control (100.00 ± 0.00%) and the other groups. No differences were observed in actin expression in the ALF model compared to the control. ZO-1 expression was unchanged at 2 h in the ALF group compared with the NS control. Each bar represents the mean ± SE (n = 3 mice per group). Insets: representative Western blots. Lane 1, NS; lane 2, 2 h after GalN/LPS administration; lane 3, 6 h after GalN/LPS administration; lane 4, 9 h after GalN/LPS administration; lane 5, LPS; lane 6, D-GalN; lane 7, anti-TNF-α-treated group; lane 8, anti-TNF-α R1-treated group; lane 9, TNF-α-treated group; lane 10, 9 h after GalN/TNF-α administration. Statistical significance was determined using a one-way ANOVA followed by the Tukey test. *P < 0.05, **P < 0.01 vs. the NS control group.

Immunohistochemical analysis revealed strong ZO-1 expression in the control groups. ZO-1 was moderately expressed in intestinal tissue 2 h after GalN/LPS or GalN/TNF-α treatment, and only traces remained 6 h after the treatments. By 9 h after the injections, it was difficult to detect positive signals for ZO-1, even in whole intestinal sections (Figure 4). ZO-1 expression was significantly increased in the two antibody-treated groups. Western blot analysis showed that ZO-1 expression decreased significantly in ALF mice, particularly 6 h and 9 h after the GalN/LPS or GalN/TNF-α injections (Figure 5). ZO-1 expression in the two antibody-treated groups was close to the normal range. These findings are consistent with the immunohistochemical results.

### Expression of ZO-1 mRNA

**Figure 6:**
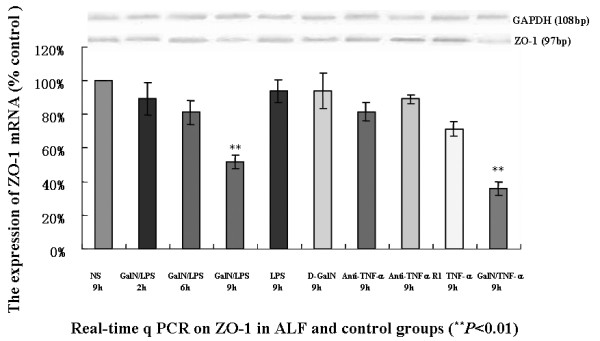
**Real-time quantitative RT-PCR for ZO-1 mRNA in the ALF groups and other control groups**. ZO-1 mRNA level was decreased at 9 h in mice with ALF. Total RNA derived from each tissue was reverse transcribed and subjected to real -time quantitative RT-PCR to evaluate ZO-1 and glyceraldehyde-3-phosphate dehydrogenase (GAPDH) mRNA levels. Results are expressed as the number of ZO-1 amplicons per 104 GAPDH amplicons. Bars represent the mean value for each group. The only significant difference in ZO-1 mRNA level between the ALF and control groups (NS: 100.00 ± 0.00%) was at 9 h. At this time, there was decrease in the level of full-length ZO-1 mRNA in the ALF mice. At all other times, there was no difference between the ALF group and the other groups, which is consistent with our immunohistochemistry results.

The ZO-1 and GAPDH RNAs used in the standard preparation were 327 bp and 335 bp long, respectively. We obtained a reasonable amplification curve, a standard curve, and a molten curve. The correlation coefficients of both standard curves were 0.999. Real-time PCR quantitative analyses showed that there were marked decreases in ZO-1 expression in ALF mice 6 h and 9 h after GalN/LPS or GalN/TNF-α treatment (*P *< 0.05) (Figure 6).

## Discussion

The intestinal mucosal barrier is composed of mucosal fluid, microvilli, epithelial mucosal cell TJs and other special structures. TJs are the most important structures in the mucosal barrier. The mechanisms responsible for SBP include cytotoxic effects and alterations in the structure of the intestinal mucosa. Altered TJ structure in active liver cirrhosis has been described [18] preciously. However, it is rarely reported the ultrastructural characteristics and TJ structrue of the intestinal mucosa in ALF and the mechanisms that link ALF with SBP are still keep unclear.

In the present study, we found that severe damage to the intestinal mucosa occurred 9 h after GalN/LPS or GalN/TNF-α treatment. Morphologic alterations to the intestinal mucosa included shedding of epithelial cells, fracturing of villi, fusion of adjacent villi, mucosal atrophy and edema. Disruption of TJs on enterocytes and damage to the mitochondria and endoplasm were also observed. The recent discovery that several polarity complexes are conserved in mammalian cells and are closely associated with TJs indicates that TJs play a vital role in establishing epithelial cell polarity [19]. Although damage to the intestinal mucosa plays a significant role in bacterial invasion of the body, the responsible mechanism remains to be elucidated.

Moreover, we observed that bacteria in the intestinal tract of ALF mice invaded the intestinal mucosa by pinocytosis at 9 h after GalN/LPS administration. It should be noted that we observed simultaneous disruptions in the integrity of the TJs. Some studies showed that the integrity of TJs is important for maintaining cellular polarity [20], a change in cell polarity may have facilitated bacterial invasion, and bacterial invasion may have occurred via the sites of disrupted TJs between intestinal mucosa epithelial cells.

ZO-1 is important for maintaining the integrity of intestinal mucosal TJs during pathological insults [21]. In our study, we found that the immunoreactive ZO-1 signal in the intestinal mucosa was significantly decreased in ALF mice, which was confirmed by the results of Western blot analysis. We also found that ZO-1 expression was significantly decreased in the intestinal tissue of human ALF patients compared with controls, which is consistent with our findings with animal models. Thus, we conclude that decreased ZO-1 expression causes TJ disruption.

To detect the key role in the mechanism of the TJ disruption and ZO-1 protein dicreased in ALF, in liver injury, inflammation involves sequential activation of signaling pathways that result in the production of pro-and anti-inflammatory mediators. Among the pro-inflammatory mediators, the TNF-α and TNF-α R1 systems play central roles in the physiological regulation of intestinal barrier function [22,23]. TNF-α and IFN-γ can induce intestinal epithelial barrier dysfunction [24]. In the present study, we found that in GalN/LPS-treated mice, TNF-a level reached a first peak at 2 h after GalN/LPS injection. We did not observe bacterial invasion of the intestinal mucosa or hepatocyte necrosis at this timepoint. The second peak occurred 9 h after the injection, at this timepoint we did observe bacterial invasion of the intestinal mucosa and hepatocyte necrosis. But bacterial invasion and hepatocyte necrosis were not observed in mice injected with either GalN or TNF-α alone. We thought that the first peak in TNF-α was the result of GalN/LPS injection, not ALF [23]. Then TNF-α was removed through degradation, whereas the second peak was released by activated macrophages, dendritic cells and kupffer cells, which was reported by Nakama T et al[24]. Moreover, some researchers have found that endotoxin (lipopolysaccharide: LPS) infection due to bacterial translocation is intimately involved in the organ failure. LPS that has translocated into portal blood binds to LPS-binding protein (LBP), was transported to the monocytes and Kupffer cells in liver sinusoids. Inflammatory cytokines such as TNF-α et al are produced and released by monocytes and Kupffer cells [25]. This study show that the bacterial invasion may play a role in the occurring of the second TNF-α peak, their relationship needs to be further investigated.

In addition, we found that the decrease in ZO-1 expression was correlated with an increase in the serum level of TNF-α. Many researchers have found that some cytokines can induce endocytosis of TJ proteins [26] and internalization of epithelial TJ proteins [27]. Reductions in levels of the tight junction protein, occludin, in intestinal epithelial cells may be caused by the production of TNF-α in mice with fulminant hepatic failure [28]. TNF-α-induced increase in Caco-2 cell TJ permeability was mediated by NF-kappa B activation. The increase in permeability was associated with NF-kappa B-dependent downregulation of ZO-1 protein expression and altered junctional localization [29-31]. In this study we found that event chronology is more important because it gives us some vital clues. The first TNF-α peak 2 h post GalN/LPS injection may induce the ZO-1 down expression and the tight junction disruption observed at 9 h. The pathophysiological processes of ALF in *vivo *is complicated, TNF-α plays an important role. TNF-α perhaps is an initiator which can induce more cytokines such as IL-6 and IFN-γ, which can aggravate liver injury and initiate the development of ALF, and the disruption of TJ intestinal. The first TNF-α peak occurred at 2 h post injection may induce the ZO-1 down expression and the tight junction disruption observed at 9 h timepoint.

There was no any damage on TJs and no decrease in the expression of ZO-1 on the mice group accepted recombinant TNF-α, the purpose of this study was to discolse the change of TJ in ALF process, a positive finding could be observed if mice accepted a high dose of recombinant TNF-α, a further study need to performed in the future.

In order to study the role of TNF-α further, we used TNF-α antibody and anti-TNFR antibody. TNF-α antibody could neutralize the quantity of TNF-α, and anti-TNFR antibody could block the combination between TNF-α and TNF-α R. When TNF-α was blockaded with anti-TNF-α IgG antibody or anti-TNF-α R1 antibody, there was a significant decrease in the mice of liver failure and no bacterial invasion or hepatocyte necrosis. These data indicate that TNF-α is an important mediator of bacterial invasion of the intestinal mucosa during ALF. Moreover, we found that a significant reduction in ZO-1 mRNA expression in ALF mice and a significant induction of ZO-1 mRNA expression in ALF mice pretreated with either anti-TNF-α IgG antibody or anti-TNF-α receptor1 antibody. These findings suggest that TNF-α downregulates ZO-1 protein expression in intestinal tissue by inhibiting ZO-1 mRNA expression.

## Conclusion

This study demonstrated the changes in intestinal mucosal morphology in mice with ALF. These changes were associated with disruption of TJ structure, changes in epithelial cell microvilli (disarrangement, distortion, and swelling or shrinkage) and mitochondrial matrices (mitochondrial swelling and disturbance of cristae). The disruption of the intestinal mucosa and consequent bacterial invasion of the body in ALF may be caused by reduced levels of the TJ-associated protein, ZO-1, the production of which was not controlled by transcription. These changes were caused by an elevated serum level of TNF-α, as they were absent when TNF-α was blocked by anti-TNF-α IgG antibody or anti-TNF-α R1 antibody. This study confirmed that TNF-α damages TJs and affects the expression of ZO-1 protein *in vivo*. TNF-α also may participate in the pathophysiological mechanism of SBP complicated to ALF. The mechanism of TNF-α-induced changes during ALF is complex and arrants further study.

## List of abbreviations

ALF: Acute liver failure; GalN: D-galactosamine; LPS: lipopolysaccharide; TJ: tight junction; ZO-1: zona occludens 1; TEM: transmission electron microscopy; TNF-α: tumor necrosis factor-a; WB: Western blot; ALT: alanine transaminase; SBP: spontaneous bacterial peritonitis; TEM: transmission electron microscopy; TNF-α R: tumor necrosis factor-a receptor; NS: normal saline.

## Competing interests

The authors declare that they have no competing interests.

## Authors' contributions

HLS and PL participated in the conception and design of the study and drafted the manuscript. HLS and SL executed the study. HLS analyzed the data. All authors have read and approved the final manuscript.

## Pre-publication history

The pre-publication history for this paper can be accessed here:

http://www.biomedcentral.com/1471-230X/9/70/prepub
